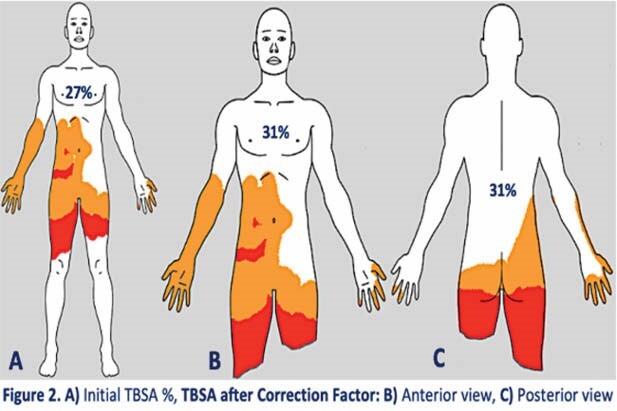# 718 Estimation of Total Body Surface Area Burns in a Bilateral Above-Knee Amputee Patient

**DOI:** 10.1093/jbcr/irad045.193

**Published:** 2023-05-15

**Authors:** Edgar Peñarrieta-Daher, Luis Treviño García, Paulina Rodríguez Villa, Jorge Morales Ortiz, Jesus Leal-Gutierrez

**Affiliations:** Hospital Universitario "Dr. José Eleuterio González", Monterrey, Nuevo Leon; Hospital Universitario "Dr. José Eleuterio González", Monterrey, Nuevo Leon; Hospital Universitario "Dr. José Eleuterio González", Monterrey, Nuevo Leon; Hospital Universitario "Dr. José Eleuterio González", Monterrey, Nuevo Leon; Hospital Universitario "Dr. José Eleuterio González", Monterrey, Nuevo Leon; Hospital Universitario "Dr. José Eleuterio González", Monterrey, Nuevo Leon; Hospital Universitario "Dr. José Eleuterio González", Monterrey, Nuevo Leon; Hospital Universitario "Dr. José Eleuterio González", Monterrey, Nuevo Leon; Hospital Universitario "Dr. José Eleuterio González", Monterrey, Nuevo Leon; Hospital Universitario "Dr. José Eleuterio González", Monterrey, Nuevo Leon; Hospital Universitario "Dr. José Eleuterio González", Monterrey, Nuevo Leon; Hospital Universitario "Dr. José Eleuterio González", Monterrey, Nuevo Leon; Hospital Universitario "Dr. José Eleuterio González", Monterrey, Nuevo Leon; Hospital Universitario "Dr. José Eleuterio González", Monterrey, Nuevo Leon; Hospital Universitario "Dr. José Eleuterio González", Monterrey, Nuevo Leon; Hospital Universitario "Dr. José Eleuterio González", Monterrey, Nuevo Leon; Hospital Universitario "Dr. José Eleuterio González", Monterrey, Nuevo Leon; Hospital Universitario "Dr. José Eleuterio González", Monterrey, Nuevo Leon; Hospital Universitario "Dr. José Eleuterio González", Monterrey, Nuevo Leon; Hospital Universitario "Dr. José Eleuterio González", Monterrey, Nuevo Leon; Hospital Universitario "Dr. José Eleuterio González", Monterrey, Nuevo Leon; Hospital Universitario "Dr. José Eleuterio González", Monterrey, Nuevo Leon; Hospital Universitario "Dr. José Eleuterio González", Monterrey, Nuevo Leon; Hospital Universitario "Dr. José Eleuterio González", Monterrey, Nuevo Leon; Hospital Universitario "Dr. José Eleuterio González", Monterrey, Nuevo Leon

## Abstract

**Introduction:**

The burn injury extension estimate is necessary for the initial IV fluid resuscitation planning, however, current Wallace rule of nines, Lund & Browder charts as well as smartphone applications are based anatomical models with full body integrity.

The purpose of this paper is to accurately estimate the TBSA burned in a bilateral asymmetric above-knee amputee patient.

**Methods:**

An amputation is defined as major amputation when the level is at or proximal ankle or wrist.

We present a case example of an 80-year-old patient with a past medical history of long-standing type 2 diabetes mellitus with inadequate metabolic control, left above-knee amputation seven years ago, as well as a contralateral asymmetric above-knee amputation seven months before hospital admission.

The patient was brought to the emergency department one hour after suffering burn injuries by fire at his shelter. Upon his arrival inhalational injury was ruled out, and at physical examination second and third-degree burns were noted circumferentially on both above-knee stumps, genitalia, right flank, lower abdomen and back, right hands and forearm, as well as left hand digits (Figure 1A-B).

Initial TBSA burned was estimated using the E-burn application yielding a 27% extension; however, this percentage would correspond to patient with full integrity of both lower limbs (Figure 2A)

**Results:**

This is the first major amputee burned patient of 285 cases admitted for in-hospital treatment at our institution from March 2020 to October 2022.

After performing an online search on MEDLINE/Pubmed (keywords: TBSA, estimation, amputated) we found only one published paper in 2020 which presented a Correction Factor (CF) formula for a hypothetical burn patient with bilateral and symmetric fore-quarter amputation.

We estimated the amputated surface area (13.7%) with e-burn, to calculate the major amputee CF published in 2020 and test it on a clinical setting. By means of the following formula (Figure 1C): CF = 100 / (100 - % of amputated body part)

CF = 100 / (100 – 13.7)

CF = 100 / (86.3) = 1.15

Finally, the initial TBSA burnt was multiplied by the CF to adjust it accordingly to major amputations of the patient, as follows: Originally estimated TBSA burnt (27%) x CF (1.15) = 31.05 % (Figure 2B-C)

**Conclusions:**

This case report serves as an example of the clinical application of the Correction Factor for major amputees burn patients which can be easily estimated with the aid of smartphone applications such as e-burn in mismatched bilateral amputations.

**Applicability of Research to Practice:**

To our knowledge this is the first clinical application of the major amputee CF, which its simplified estimation by means of E-burn could be of use for burn centers with higher volume.